# Immune Subtypes Characterization Identifies Clinical Prognosis, Tumor Microenvironment Infiltration, and Immune Response in Ovarian Cancer

**DOI:** 10.3389/fmolb.2022.801156

**Published:** 2022-03-21

**Authors:** Weihong Lu, Fei Zhang, Xiaolin Zhong, Jinhua Wei, Hongyang Xiao, Ruiqin Tu

**Affiliations:** ^1^ Zhongshan Hospital, Fudan University (Xiamen Branch), Xiamen, China; ^2^ Zhongshan Hospital, Fudan University, Shanghai, China

**Keywords:** ovarian cancer, immune subtype, prognosis, tumor microenvironment, immunotherapy

## Abstract

**Objective:** Because of the modest immunotherapeutic response among ovarian carcinoma (OC) patients, it is significant to evaluate antitumor immune response and develop more effective precision immunotherapeutic regimens. Here, this study aimed to determine diverse immune subtypes of OC.

**Methods:** This study curated the expression profiles of prognostic immunologically relevant genes and conducted consensus clustering analyses for determining immune subtypes among OC patients in TCGA cohort. With Boruta algorithm, characteristic genes were screened for conducting an immune scoring system through principal component analysis algorithm. The single-sample gene set enrichment analysis and ESTIAMTE methods were adopted for quantifying the immune infiltrates and responses to chemotherapeutic agents were estimated with pRRophetic algorithm. Two immunotherapeutic cohorts were used for investigating the efficacy of immune score in predicting therapeutic benefits.

**Results:** Two immune subtypes were conducted among 377 OC patients. Immune subtype 2 was characterized by worse clinical prognosis, more frequent genetic variations and mutations, enhanced immune infiltrates, and increased expression of MHC molecules and programmed cell death protein 1/programmed death ligand 1 (PD-1/PD-L1). In total, 30 prognosis-relevant characteristic immune subtype–derived genes were identified for constructing the immune score of OC patients. High immune score was linked with more dismal prognosis, decreased immune infiltrations, and expression of MHC molecules. High immune score presented favorable sensitivity to doxorubicin and vinorelbine and reduced sensitivity to cisplatin. In addition, immune score possessed the potential in predicting benefits from anti–PD-1/PD-L1 therapy.

**Conclusion:** Collectively, our findings propose two complex and diverse immune subtypes of OC. Quantitative assessment of immune subtypes in individual patients strengthens the understanding of tumor microenvironment features and promotes more effective immunotherapeutic regimens.

## Introduction

Ovarian carcinoma (OC) is the leading cause of deaths among females with gynecological malignant tumors (Kuroki and Guntupalli, 2020). Approximately 90% OC patients are of epithelial cell origin (Lheureux et al., 2019). Currently, cytoreductive surgery, platinum-relevant chemotherapy, targeted therapy, and immunotherapy remain the major therapeutic regimens (Baci et al., 2020). Nevertheless, OC possesses the highest mortality among gynecological malignancies because most patients present advanced and metastatic tumors at the time of diagnosis (Song et al., 2020). Although 80% of newly diagnosed patients respond to the first-line therapy containing cytoreductive surgery and platinum-based chemotherapy, approximately 75% with advanced stages experience relapse that represents the main characteristics of OC (MacGregor et al., 2019). Moreover, resistance usually occurs, which contributes to a 5-year survival rate of <50% among females <65 years old and <30% among females >65 years old (Natoli et al., 2020). Poly(ADP-ribose) polymerase (PARP) inhibitor has emerged as the first targeted agent as maintenance treatment in platinum-sensitive relapsed patients, which presents the significant association with prominent clinical benefit (Jiang et al., 2020). Unfortunately, PARP inhibitor is merely restricted to 10% patients who have BRCA mutations, and the therapeutic effects are restricted because of distinct resistance phenomenon (Mirza et al., 2020). Hence, novel therapeutic regimens and markers remain urgently required.

Immunotherapy emerges as a prospective therapeutic modality, possessing well specificity, long-term effects, and few side effects. The response rate of immune checkpoint blockade therapy is only 15% for OC patients because of widespread heterogeneity such as clinicopathologic characteristics, molecular features, immune microenvironment, and so on (Gao et al., 2020). Hence, precise identification of underlying benefits in patients remains critical for improvement of the immunotherapy considering OC heterogeneity. Nevertheless, the heterogeneity of immune microenvironment of OC remains indistinct. At present, consensus signature remains scarce for inferring the immune activity in OC and stratifying OC patients accordingly. Although numerous prognostic signatures have been proposed for stratifying OC patients, they cannot estimate the antitumor immune activity (Hao et al., 2018). Here, this study conducted two diverse immune subtypes with different immune infiltrates and immune responses and developed an immune scoring system in OC patients, which strengthened an in-depth comprehending of tumor microenvironment features and triggered effective immunotherapeutic regimens.

## Materials and Methods

### Data Extraction and Processing

Molecular data of 377 patients with a diagnosis of OC were curated from The Cancer Genome Atlas (TCGA; http://cancergenome.nih.gov/) project. Transcriptome profiles (HTSeq-FPKM) and clinical data of this TCGA-OC dataset were downloaded from the GDC data portal (https://portal.gdc.cancer.gov). Ensemble IDs were transformed to gene symbols, and FPKM values were converted to transcript per million (TPM). Publicly available Affymetrix microarrays and follow-up information of 107 OC patients were harvested from the Gene Expression Omnibus (http://www.ncbi.nlm.nih.gov/geo/) with an accession number of GSE26193 (Gentric et al., 2019), which were utilized for external verification. GISTIC2.0 was adopted for analyzing copy number variation (CNV) data retrieved from TCGA, and significantly deleted or amplified genes were determined at *q* < 0.05 and fragments that had >0.1 deletion or amplification length. Single-nucleotide polymorphism (SNP) data of 436 OC patients stored in Mutation Annotation Format (MAF) files was retrieved from TCGA *via* the GDC Data Portal, which was analyzed with Maftools package (Mayakonda et al., 2018). In total, 200 immunologically relevant genes were collected from the Molecular Signatures Database (MSigDB; http://www.broadinstitute.org/msigdb) (Liberzon et al., 2015). Data were analyzed with R software (version 3.6.1) and available packages.

### Screening of Prognostic Immunologically Relevant Genes

Univariate Cox proportional hazard regression analyses were implemented for determining the associations of gene expression with overall survival (OS) of OC patients. Genes under *p* < 0.05 were considered as prognostic immunologically relevant genes. Hazard ratios (HRs) derived from univariate Cox regression analyses were utilized for determining protective (HR <1) or risk genes (HR >1).

### Construction of Immune Subtypes by Consensus Clustering Analyses

Immune subtypes were identified through ConsensusClusterPlus package (Wilkerson and Hayes, 2010). A consensus matrix was first conducted via consensus clustering analyses for classifying OC specimens. Following PAM algorithm and 1-Pearson correlation coefficient as metric distanced 500 bootstraps were presented, each involving 80% of OC patients in TCGA cohort, the number of clusters was set at 2 to 10, and consensus clustering was adopted for classifying the prognostic immunologically relevant genes. Consistency matrix and consistency cumulative distribution function were adopted for determining the best classification. This consensus clustering was verified in the GSE26193 cohort.

### Quantification of Hallmark Pathways by Gene Set Variation Analysis

The 50 hallmark gene sets were curated from the MSigDB project. Single-sample gene set enrichment analyses (ssGSEA) were conducted for calculating the enrichment scores of hallmark gene sets utilizing Gene Set Variation Analysis package (Hänzelmann et al., 2013). Hierarchical clustering of hallmark pathway enrichment scores was presented with pheatmap package.

### Quantification of Immune Cell Infiltrations

The gene sets that represented diverse infiltrating immune cell subpopulations were curated from Bindea et al. (2013). Thereafter, ssGSEA was conducted for estimating the abundance of immune cell subpopulations containing innate and adaptive immune cells in accordance with the expression of reference genes from transcriptomic profiling. Estimation of STromal and Immune cells in MAlignant Tumor tissues using Expression data (ESTIMATE) algorithm (Yoshihara et al., 2013) was adopted for estimating the presence of stromal and immune cells within the tumor microenvironment through calculating specific mRNA expression markers. Immune scores that represented the infiltrations of immune cells as well as stromal scores that represented the presence of stroma in tumor tissues were separately calculated on the basis of the mRNA expression profiling. In addition, tumor purity was estimated *via* integrating stromal and immune scores. Tumor mutation burden (TMB) was calculated in each specimen in accordance with the number of variants/the length of exons utilizing Perl scripts on the basis of JAVA8 background.

### Quantification of Immune Score by Principal Component Analyses

The mRNA expression values in diverse immune subtypes were analyzed with limma package (Ritchie et al., 2015). In accordance with log2 |fold-change| >1 and false discovery rate (FDR) < 0.0001, immune subtype–derived genes were screened. With univariate Cox regression models, prognosis-relevant immune subtype–derived genes were identified with *p* < 0.05. Thereafter, Boruta feature importance analyses were conducted for feature selection with Boruta package (Shi et al., 2019). The expression profiling of the finally identified genes was curated for presenting principal component analysis (PCA). Moreover, principal component 1 (PC1) and PC2 were extracted and acted as immune score. The immune score was calculated following the formula: immune score = ∑(PC1i + PC2i), in which *i* meant the expression of the finally identified prognosis-relevant characteristic immune subtype–derived genes.

### Function Annotation Analyses

The clusterProfiler package (Yu et al., 2012) was implemented for presenting Kyoto Encyclopedia of Genes and Genomes (KEGG) and Gene Ontology (GO) enrichment analyses. GO terms comprised three categories: biological process, cellular component, and molecular function.

### Prediction of the Benefits From Chemotherapy

Six commonly applied chemotherapeutic agents (paclitaxel, etoposide, gemcitabine, doxorubicin, vinorelbine, and cisplatin) were selected from the Genomics of Drug Sensitivity in Cancer (GDSC) project (www.cancerRxgene.org) (Yang et al., 2013), the largest publicly available pharmacogenomics database. The predictive procedure was conducted with pRRophetic package (Geeleher et al., 2014). The half-maximal inhibitory concentration (IC_50_) was determined utilizing ridge regression analyses. On the basis of GDSC training set, the predictive accuracy was evaluated with 10-fold cross-verification.

### Collection of Genomic and Clinical Data of Immunotherapy Cohorts

Two immunotherapy cohorts were collected in our study: metastatic melanoma treated with anti–programmed cell death protein 1 (PD-1) inhibitors from Liu et al. (2019) and locally advanced or metastatic urothelial carcinoma treated with anti–programmed death ligand 1 (PD-L1) inhibitor atezolizumab from the IMvigor210 cohort (Mariathasan et al., 2018). For the cohortof Liu et al. (2019), following normalization with limma package, the FPKM data were converted into TPM values across specimens. For IMvigor210 cohort, mRNA expression profiling and prognostic information were curated from http://research-pub.Gene.com/imvigor210corebiologies with the Creative Commons 3.0 License. Raw count data were standardized with DEseq2 package, which was converted to TPM.

### Statistical Analyses

All the computational and statistical analyses were implemented with R software (https://www.r-project.org/). Through t-distributed stochastic neighbor embedding (t-SNE)–based method, the subtype assignments were verified utilizing the mRNA expression profiling of immune genes. Univariate Cox proportional hazards regression models were utilized for estimating the HRs. OS, disease-specific survival (DSS), and progression-free interval (PFI) analyses were presented utilizing Kaplan–Meier methods, and comparisons between groups were presented through log-rank tests. Comparisons between two groups with normally distributed variables were conducted with unpaired Student *t* test. In addition, two groups with non–normally distributed variables were compared with Mann–Whitney *U* test. A two-tailed *p* < 0.05 indicates statistical significance.

## Results

### Characterization of Two Diverse Immune Subtypes of OC

**FIGURE 1:**
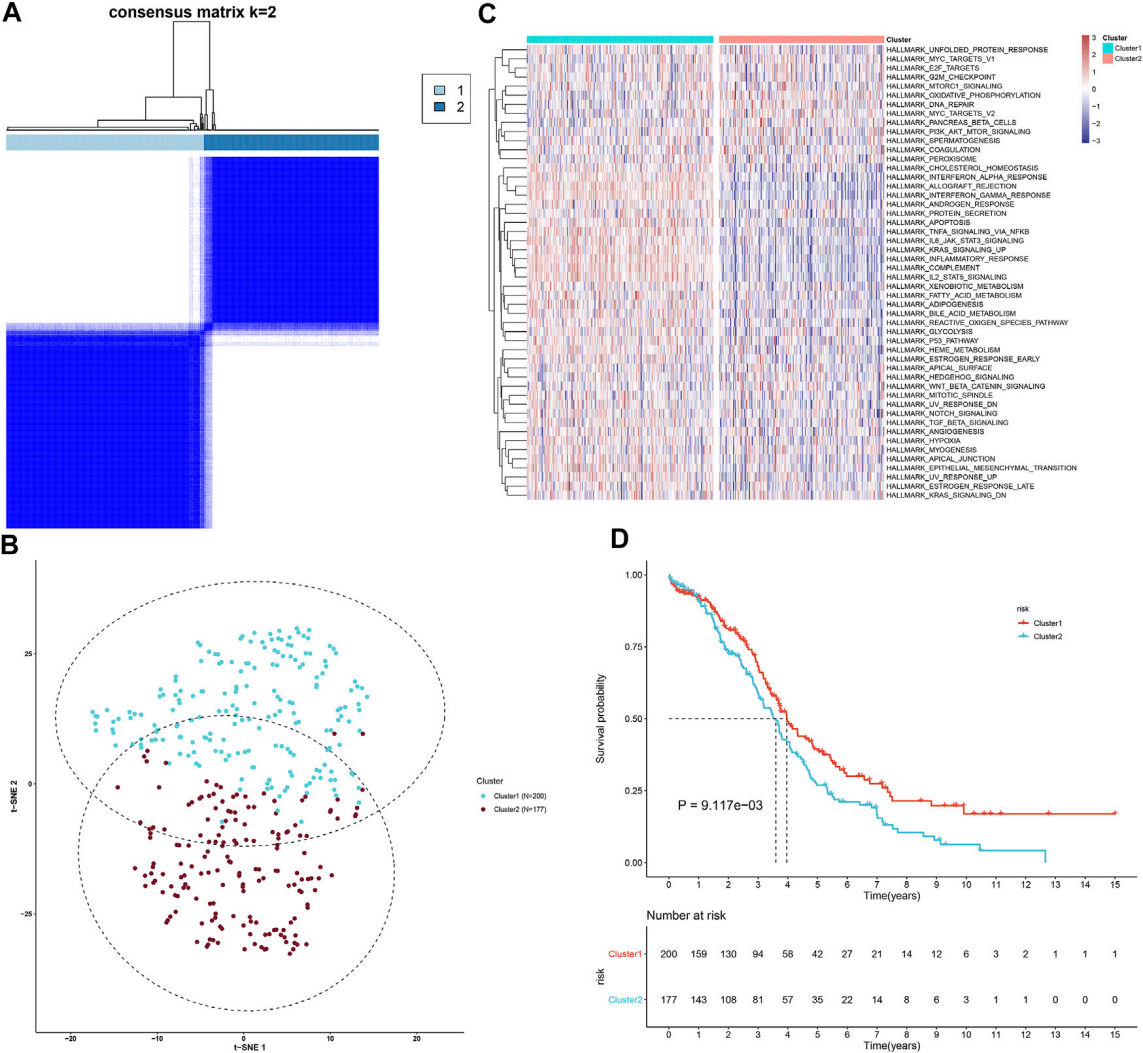
Characterization of diverse immune subtypes of OC patients. **(A)** Heatmap depicting the sample clustering when consensus *k* = 2 in accordance with the expression profiling of prognostic immunologically relevant genes. **(B)** The t-SNE analyses visualizing two discrete immune subtypes with expression matrix of prognostic immunologically relevant genes. **(C)** Heatmap showing the activation difference in hallmark gene sets between two immune subtypes. **(D)** Kaplan–Meier analyses for the survival difference between two immune subtypes.

This study downloaded mRNA expression profiling of immunologically relevant genes of OC specimens from TCGA cohort. Utilizing univariate Cox regression models, we first screened 26 prognostic immunologically relevant genes across OC patients (Table 1). Through consensus clustering analyses, OC patients were clustered in accordance with the expression profiling of prognostic immunologically relevant genes. The stability of this clustering was evaluated from *k* = 2–10. As a result, *k* = 2 was the optimal choice (Figure 1A). Thus, two immune subtypes were identified as immune subtype 1 (n = 200) and immune subtype 2 (n = 177) across OC patients. Our t-SNE analyses suggested that OC specimens were clearly separated into two diverse subtypes in accordance with the expression matrix of prognostic immunologically relevant genes (Figure 1B). We further investigated the biological discrepancy between immune subtypes. Compared with immune subtype 1, oncogenic pathways, such as MYC, E2F, DNA repair, and PI3K-Akt-mTOR signaling, presented prominent activation in immune subtype 2 (Figure 1C). In addition, immune activation pathways, such as allograft rejection, tumor necrosis factor α signaling via nuclear factor κB, interleukin 6 (IL6)–JAK–STAT3 signaling, inflammatory response, complement, and IL2–STAT5 signaling, had higher activation in immune subtype 1 than immune subtype 2. Kaplan–Meier analyses uncovered the patients in immune subtype 2 with more dismal clinical outcomes (Figure 1D).

**TABLE 1:** Identification of prognostic immunologically relevant genes across OC patients.

Gene	HR	HR.95L	HR.95H	*p* Value
ATP2B1	1.21901	1.00119	1.48422	0.04863
AXL	1.147504	1.00359	1.31206	0.04418
BDKRB1	1.317952	1.01262	1.71535	0.04005
C5AR1	1.189023	1.05108	1.34507	0.00593
CCR7	0.802639	0.6763	0.95259	0.01188
CLEC5A	1.193255	1.05213	1.35331	0.00594
CXCL10	0.92617	0.87058	0.98531	0.01516
CXCL11	0.87755	0.81709	0.94249	0.00034
CXCL9	0.902788	0.84517	0.96434	0.00237
FPR1	1.128597	1.00823	1.26333	0.03552
GCH1	0.825432	0.69403	0.98172	0.03012
ITGB8	1.177166	1.0391	1.33358	0.01039
LAMP3	0.879158	0.79961	0.96662	0.00778
LCK	0.860916	0.74519	0.99462	0.04203
LTA	0.735439	0.58439	0.92553	0.0088
NFKB1	1.20125	1.00195	1.44019	0.04759
PTGER2	1.165763	1.05511	1.28802	0.00258
RAF1	1.264731	1.00908	1.58515	0.04151
RNF144B	0.870796	0.77745	0.97535	0.01679
SCN1B	1.32173	1.11378	1.56851	0.0014
SELL	0.867218	0.7711	0.97532	0.01746
SELENOS	0.796482	0.63984	0.99147	0.04169
SLAMF1	0.628884	0.4579	0.86372	0.00417
SLC31A2	1.330856	1.01141	1.7512	0.04125
SLC7A1	1.197531	1.03083	1.39119	0.01843
STAB1	1.159254	1.03486	1.29861	0.01072

### Immune Subtypes With Diverse Genomic Variations Across OC

**FIGURE 2:**
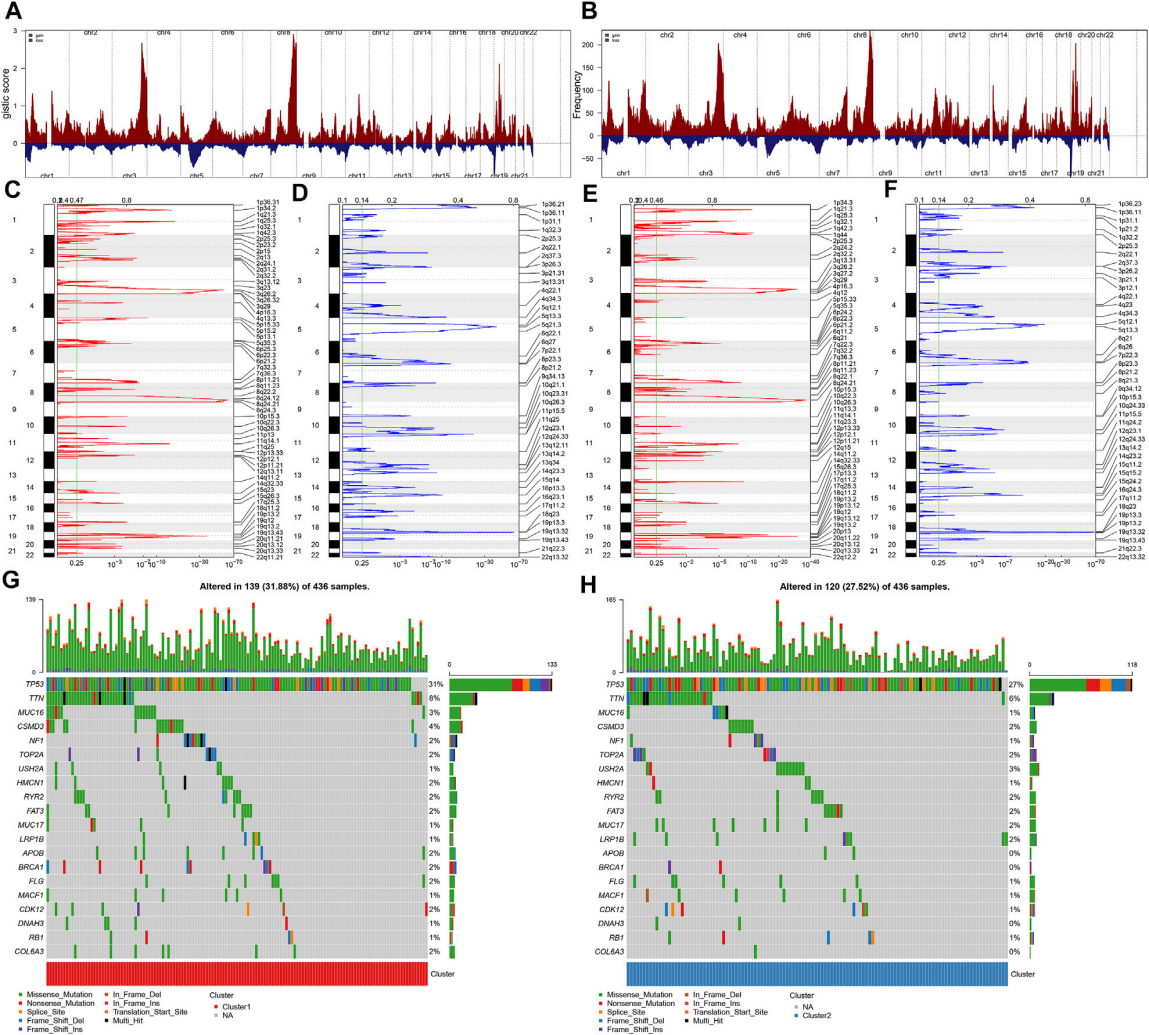
Immune subtypes with diverse genomic variations across OC. **(A,B)** Landscape of the GISTIC score and frequency of significant amplifications (red) and deletions (blue) across OC specimens with GISTIC2.0 software. Red meant amplification, whereas blue meant deletion. **(C,D)** Prominently amplified and deleted fragments in immune subtype 1. **(E,F)** Prominently amplified and deleted fragments in immune subtype 2. The *q* value and score calculated by GISTIC2.0 for variations (*x* axis) were depicted corresponding to the genome locations (*y* axis). The dotted lines indicate centromeres. The green lines meant *q* value threshold to determine significantly mutated genes. **(G,H)** Oncoprint of the somatic mutation landscape across OC specimens from **(G)** immune subtype 1 and **(H)** immune subtype 2.

With GISTIC2.0, genes with prominent amplifications or deletions were investigated across OC specimens. Figures 2A,B display genes with prominent amplifications and deletions within each fragment. There were 2,681 significant genes (*q* < 0.05) harboring 59 amplified fragments as well as 4,005 significant genes (*q* < 0.05) within 42 deleted fragments across OC specimens from immune subtype 1 (Figures 2C,D). Meanwhile, 3,773 genes had amplifications within 56 fragments, whereas 5,479 genes had deletions harboring 43 fragments across OC specimens from immune subtype 2 (Figures 2E,F). This indicates the prominent difference in CNVs between immune subtypes, with more frequent CNVs in immune subtype 2. We also investigated the distribution of SNPs across OC specimens. In total, 139 OC samples had genetic mutations in immune subtype 1 (Figure 2G), whereas 120 had mutations in immune subtype 2 (Figure 2H), indicating that samples in immune subtype 1 presented higher probability of genetic mutations. Both in two subtypes, TP53 was the most frequently mutated gene, followed by MUC16. In addition, missense mutation was the leading mutated type.

### Two Immune Subtypes With Distinct Immune Infiltrates and Immune Response

**FIGURE 3:**
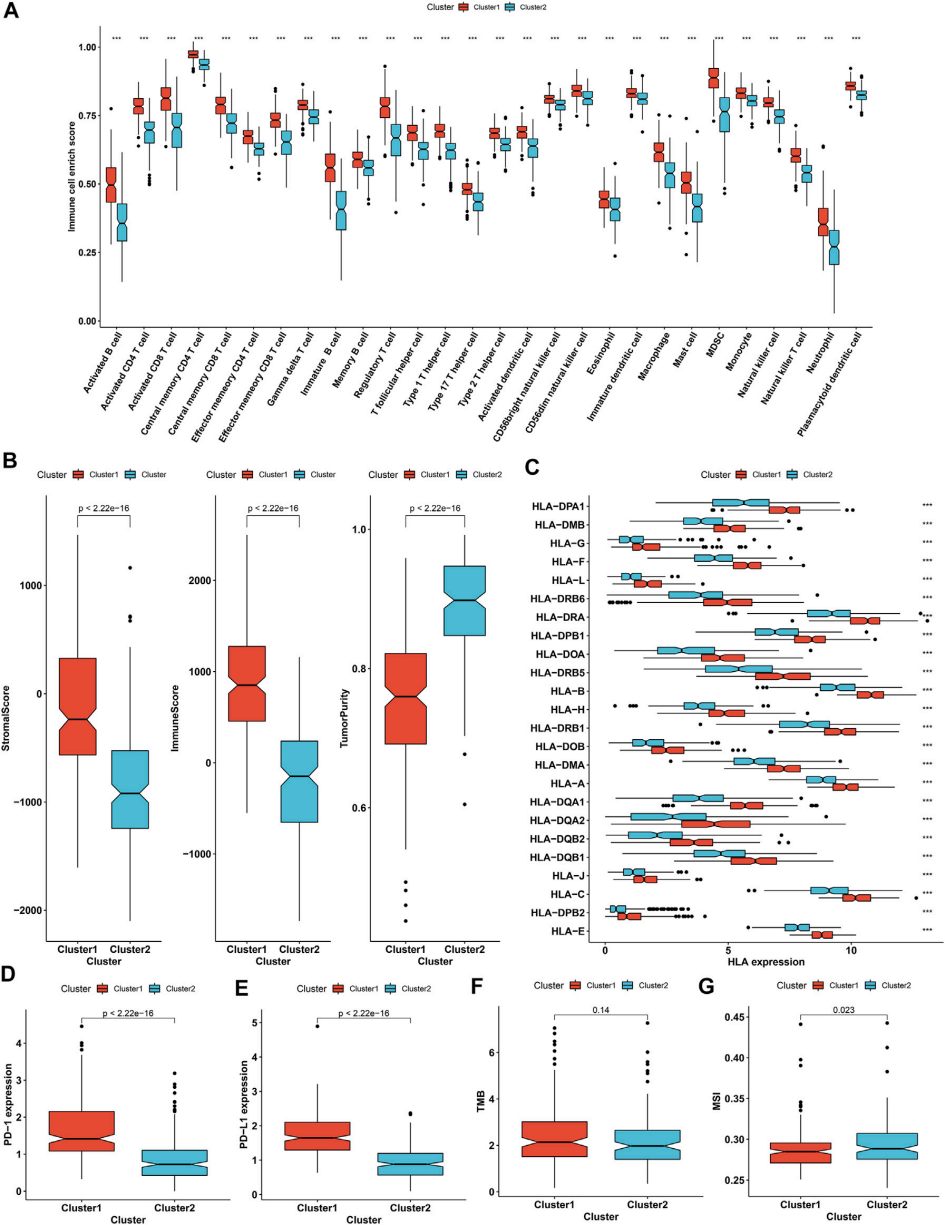
Two immune subtypes with disparate immune infiltrates and immune response across OC patients. **(A)** The ssGSEA identifying the difference in immune infiltrates between immune subtypes 1 and 2. **(B)** ESTIMATE inferring the difference in stromal and immune scores and tumor purity between two immune subtypes. **(C)** Comparison of the difference in expression of MHC molecules between two immune subtypes. **(D,E)** Box plots presenting the difference in expression of PD-1 and PD-L1 between two immune subtypes. **(F,G)** Estimation of the difference in TMB and MSI scores between two immune subtypes. ***p < 0.001.

With ssGSEA algorithm, we estimated the infiltrations of immune cell subpopulations across OC specimens. In comparison to immune subtype 2, most immune cell subpopulations presented remarkably enhanced infiltrations in immune subtype 1, containing activated, immature, and memory B cells; activated, central memory, and effector memory CD4 T cells; activated, central memory, and effector memory CD8 T cells; gamma delta, and regulatory T cells; T follicular, type 1, type 2, and type 17 helper cells (Th1, Th2, Th17); activated, immature, and plasmacytoid dendritic cells; CD56bright and CD56dim natural killer cells; natural killer cells; natural killer T cells; eosinophils; macrophages; mast cells; myeloid-derived suppressor cells; monocytes; and neutrophils (Figure 3A). In accordance with ESTIMATE algorithm, we inferred that immune subtype 1 showed increased stromal and immune score as well as reduced tumor purity than immune subtype 2 (Figure 3B). In addition, most MHC molecules had higher mRNA expression in immune subtype 1 than 2 (Figure 3C). PD-1/PD-L1 signaling is responsible for tumor immune escape, which acts as the major immune checkpoints in cancer immunotherapy. Remarkably enhanced expression of PD-1 and PD-L1 was investigated in immune subtype 1 than 2 (Figures 3D,E). TMB status represents a potential immune response predictor, and we investigated that immune subtype 1 presented increased TMB score compared with immune subtype 2 (Figure 3F). In addition, we noticed the reduced microsatellite instability (MSI) score in immune subtype 1 (Figure 3G). Above evidences uncovered that two immune subtypes presented diverse immune infiltrates and immune responses. Especially, patients in immune subtype 1 might have better chance to respond to immunotherapy.

### Identification of Immune Subtype-Derived Genes

**FIGURE 4:**
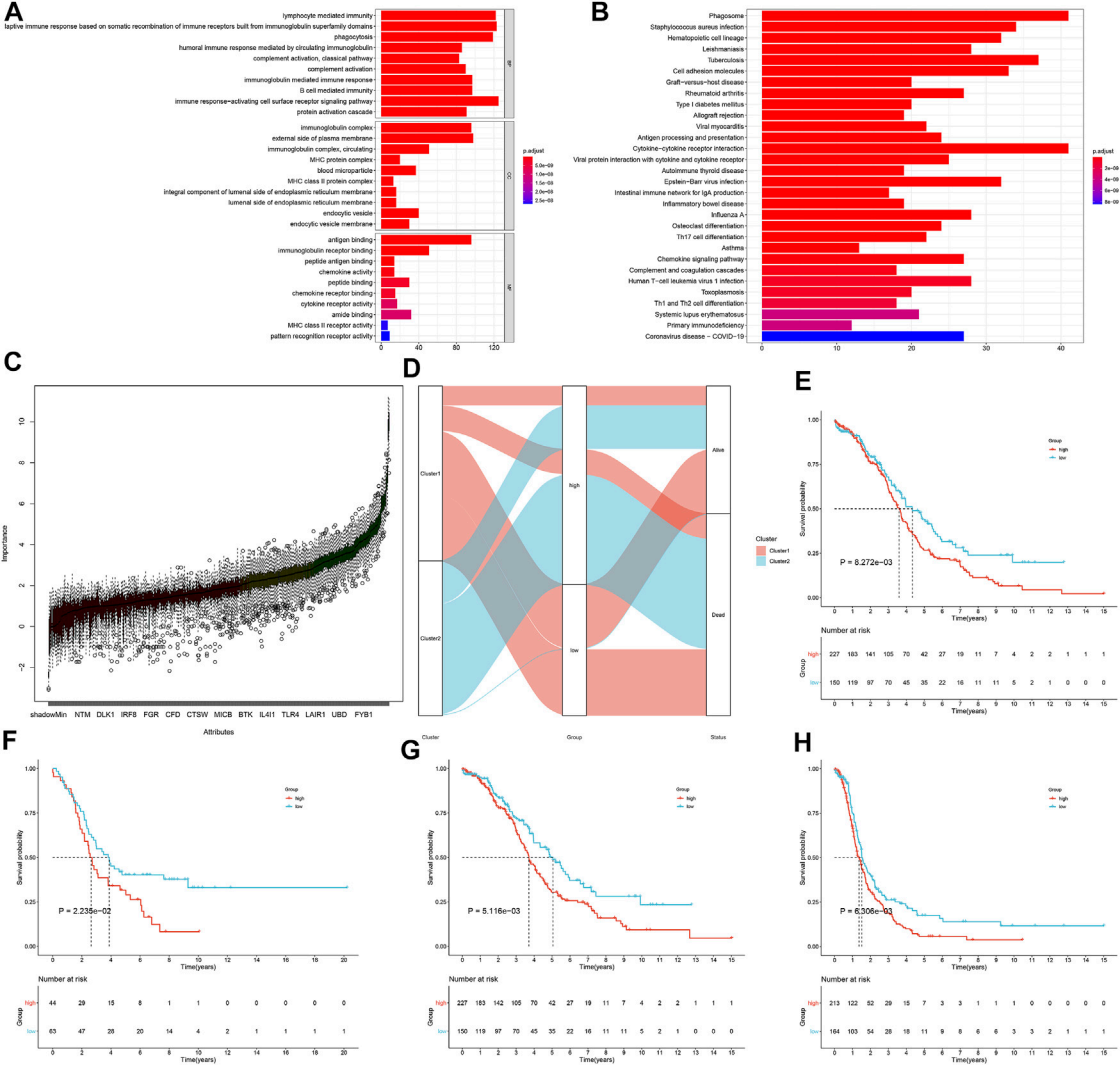
Development of an immune scoring system for OC. **(A)** GO enrichment results of immune subtype–derived genes. **(B)** KEGG pathway enrichment results of immune subtype–derived genes. **(C)** Identification of characteristic immune subtype–derived genes with Boruta feature importance analyses. **(D)** Alluvial diagram depicting the connections of immune subtypes, immune score, and survival status. **(E)** Kaplan–Meier analyses for the OS discrepancy in OC patients with high and low immune score in TCGA cohort. **(F)** Verification of the OS difference between high and low immune score patients in the GSE26193 dataset. **(G,H)** Kaplan–Meier analyses for the DSS and PFI difference in OC patients with high or low immune score in TCGA cohort.

In accordance with log2 |fold-change| >1 and FDR <0.0001, we screened 499 immune subtype–derived genes (Supplementary Table S1). Thereafter, biological significance of these immune subtype–derived genes was investigated through GO and KEGG annotation analyses. Our investigation results demonstrate that immune subtype–derived genes mainly participated in modulating immunity-relevant biological processes (such as lymphocyte mediated immunity, adaptive immune response, humoral immune response mediated by circulating immunoglobulin, complement activation, complement activation, immunoglobulin mediated immune response, B cell–mediated immunity, and immune response-activating cell surface receptor signaling pathway), cellular components (such as immunoglobulin complex, immunoglobulin complex, MHC protein complex, and MHC class II protein complex), and molecular functions (such as antigen binding, immunoglobulin receptor binding, peptide antigen binding, chemokine activity, chemokine receptor binding, cytokine receptor activity, MHC class II receptor activity, and pattern recognition receptor activity; Figure 4A). In addition, immunity-relevant pathways were enriched by immune subtype–derived genes such as cell adhesion molecules, allograft rejection, antigen processing and presentation, cytokine-cytokine receptor interaction, intestinal immune network for immunoglobulin A production, Th17 cell differentiation, chemokine signaling pathway, complement and coagulation cascades, human T-cell leukemia virus 1 infection, Th1 and Th2 cell differentiation, and primary immunodeficiency (Figure 4B). Above data confirmed the critical roles of immune subtype–derived genes in modulating tumor immunity.

### Development of an Immune Scoring System for OC

With Boruta feature importance analyses, we identified 119 characteristic immune subtype–derived genes (Figure 4C and Supplementary Table S2). Among them, univariate Cox regression models uncovered that 30 characteristic immune subtype–derived genes presented distinct correlations to OC prognosis (Table 2). On the basis of the above genes, an immune scoring system was developed through PCA algorithm. Alluvial diagram depicted the connections of immune subtypes, immune score, and survival status (Figure 4D). Survival analyses uncovered that patients with high immune score presented more dismal clinical prognosis than those with low immune score (Figure 4E). The predictive efficacy of this immune score in OC prognosis was externally confirmed in the GSE26193 dataset (Figure 4F). Moreover, high immune score was indicative of poorer DSS (Figure 4G) and PFI (Figure 4H) for OC patients.

**TABLE 2:** Prognosis-relevant characteristic immune subtype–derived genes in OC.

Gene	HR	HR.95L	HR.95H	*p* Value
GBP5	0.98209	0.96569	0.99877	0.03545
CXCL11	0.98853	0.98237	0.99472	0.000295
CXCL10	0.99851	0.99751	0.99951	0.003436
CD2	0.98196	0.96669	0.99747	0.022801
IL2RG	0.99208	0.98468	0.99954	0.037483
CXCL9	0.9941	0.99014	0.99807	0.003616
GBP4	0.98868	0.98047	0.99696	0.00749
FCGR2A	1.00657	1.00143	1.01172	0.012093
SLAMF7	0.97624	0.95388	0.99913	0.04196
TRAC	0.99069	0.9826	0.99884	0.02522
CD163	1.01292	1.00531	1.02059	0.000845
ANKRD22	0.95146	0.91092	0.99381	0.025132
TAP1	0.99627	0.99405	0.99849	0.001017
SELL	0.97426	0.95262	0.99638	0.022841
GBP1P1	0.95934	0.93987	0.97921	7.21E-05
SIGLEC1	1.01404	1.00027	1.028	0.045651
GBP1	0.99713	0.99453	0.99974	0.030903
CD38	0.92642	0.87223	0.98399	0.012961
EPB41L3	1.04629	1.01447	1.0791	0.004082
HLA-B	0.99987	0.99974	0.99999	0.039989
CXCL13	0.9882	0.97945	0.99702	0.008825
WARS	0.99727	0.99546	0.99909	0.003241
ETV7	0.97791	0.96095	0.99516	0.012303
OR2I1P	0.96662	0.93594	0.99831	0.039137
STAT1	0.99828	0.99681	0.99975	0.02183
USP30-AS1	0.93008	0.8837	0.9789	0.005486
ZBP1	0.95285	0.91443	0.99288	0.021422
BATF2	0.97058	0.94958	0.99205	0.007472
UBD	0.9877	0.97632	0.99922	0.036419
C2	0.99526	0.99131	0.99923	0.019289

### This Immune Score Predicts Immune Infiltrates and Immune Responses of OC

**FIGURE 5:**
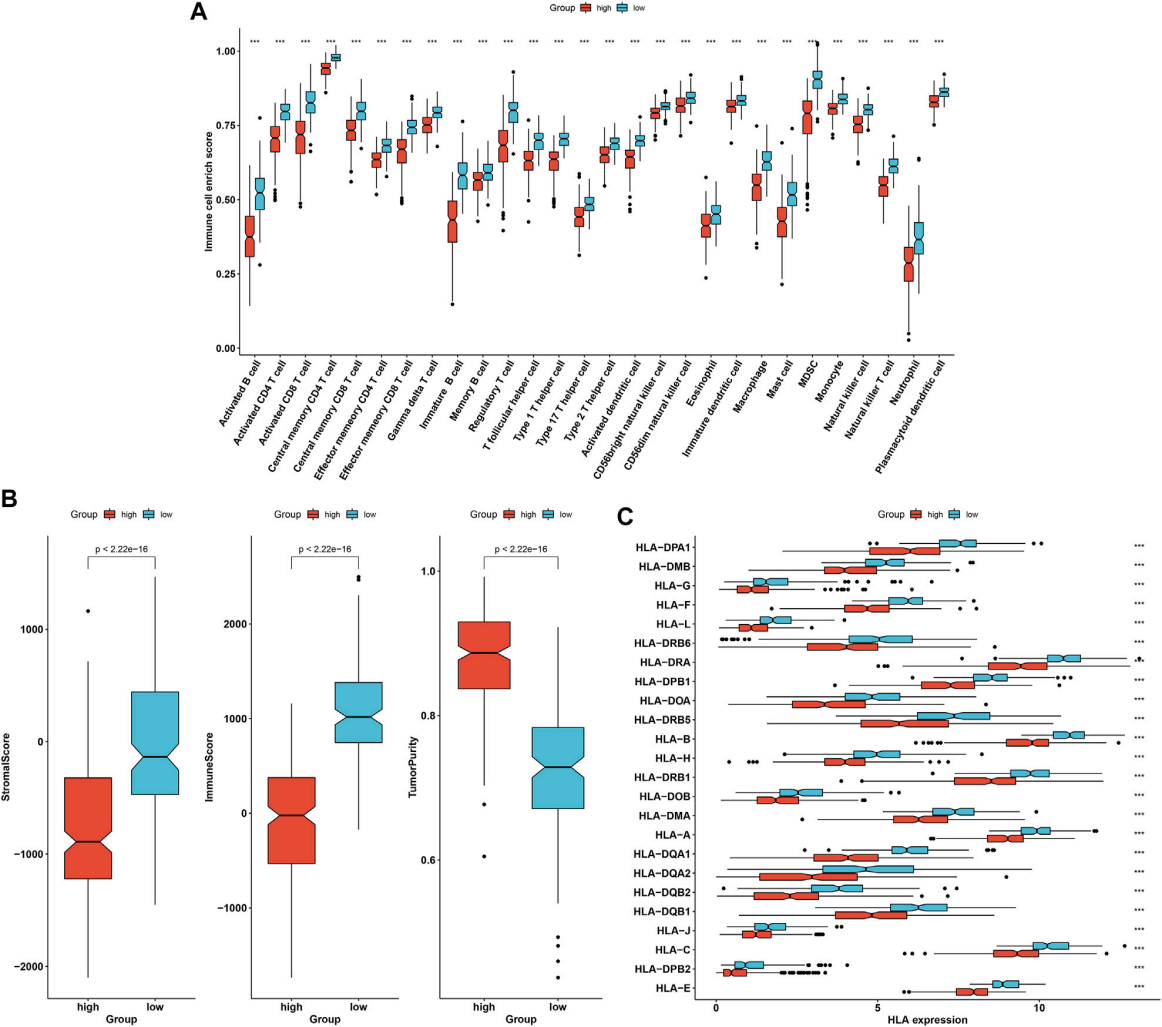
This immune score predicts immune infiltrates and immune responses of OC. **(A)** Distribution of immune infiltrates between high and low immune score OC patients with ssGSEA algorithm. **(B)** The difference in stromal and immune score and tumor purity between high and low immune score groups. **(C)** Comparison of the expression of MHC molecules in two groups. ***p < 0.001.

Further analyses demonstrate that OC specimens with low immune score presented the characteristics of enhanced infiltrations of most immune cell subpopulations (Figure 5A). In addition, there were increased stromal and immune scores as well as weakened tumor purity in low immune score group (Figure 5B). In Figure 5C, most MHC molecules had enhanced expression for patients with low immune score. Above data indicate that the immune score possessed the potential in estimating immune infiltrates and immune responses of OC.

### This Immune Score Associates With Chemotherapeutic Responses

**FIGURE 6:**
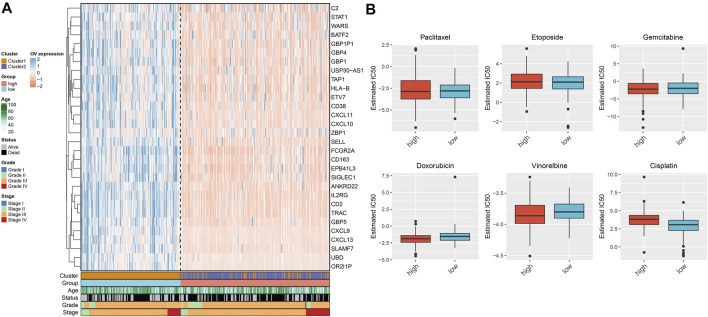
This immune score is associated with chemotherapeutic responses of OC patients. **(A)** Heatmap visualizing the expression distributions of prognosis-relevant characteristic immune subtype–derived genes across OC patients. **(B)** The difference in estimated IC_50_ values of chemotherapeutic agents (paclitaxel, etoposide, gemcitabine, doxorubicin, vinorelbine, and cisplatin) in high and low immune score groups.

We investigated the expression of prognosis-relevant characteristic immune subtype–derived genes across OC specimens. Figure 6A depicts that most genes presented enhanced expression both in immune subtype 2 and high immune score group. In addition, IC_50_ values of commonly applied chemotherapeutic agents were determined across OC patients. No significant difference in estimated IC_50_ values of paclitaxel, etoposide, and gemcitabine was detected between immune score groups (Figure 6B). Higher IC_50_ values of doxorubicin and vinorelbine as well as reduced IC_50_ value of cisplatin were observed in low immune score group. This indicates that patients with high immune score presented higher sensitivity to doxorubicin and vinorelbine as well as lower sensitivity to cisplatin.

### This Immune Score Acts as a Predictor of Immunotherapeutic Benefits

**FIGURE 7:**
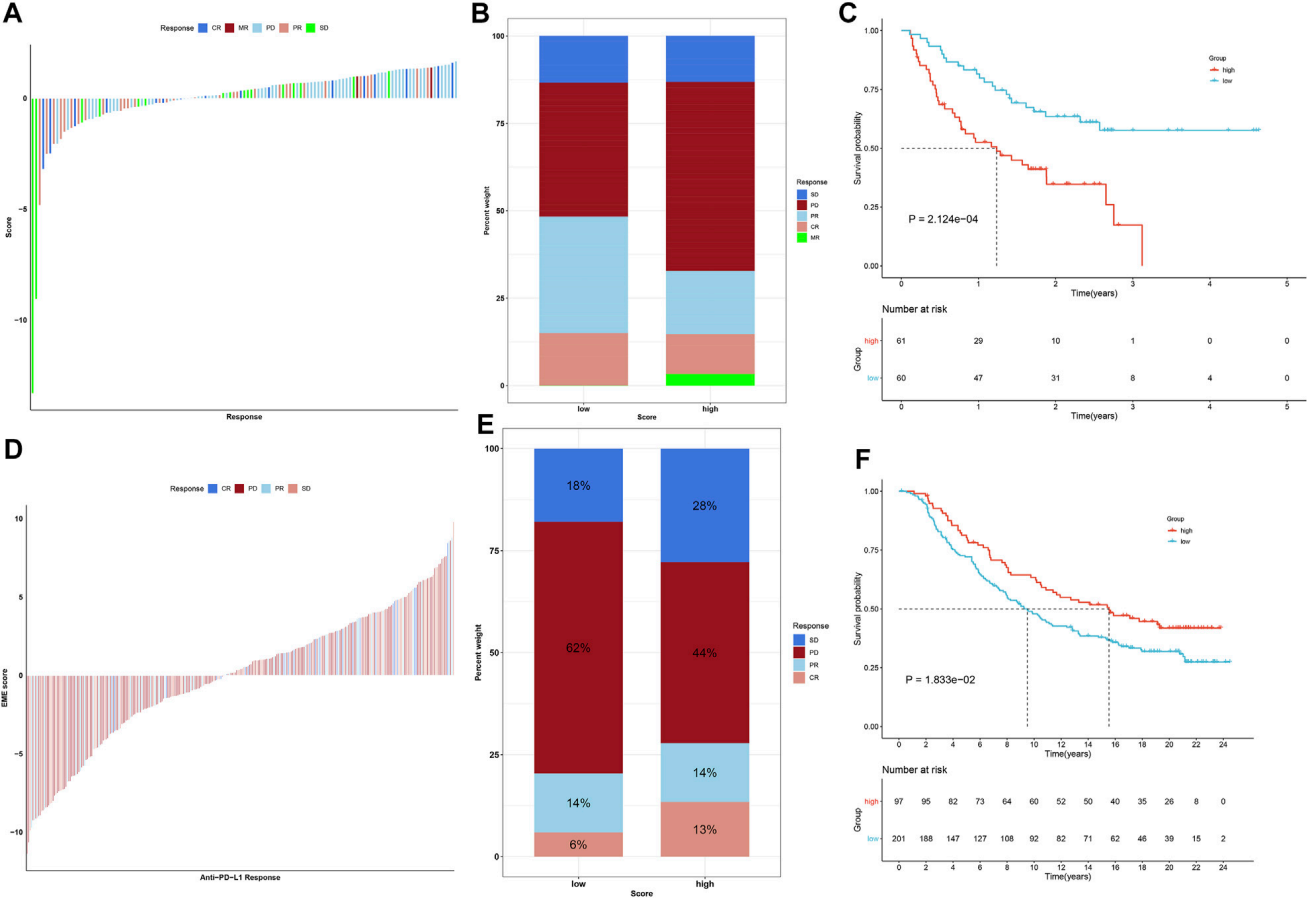
This immune score acts as a predictor of immunotherapeutic responses. **(A)** Landscape of therapeutic responses to anti–PD-1 inhibitor in the Liu et al. cohort. **(B)** The fractions of responses to anti–PD-1 immunotherapy in high and low immune score groups in the Liu et al. cohort. **(C)** Kaplan–Meier analyses for high and low immune score patients in the Liu et al. cohort. **(D)** Landscape of therapeutic responses to anti–PD-L1 inhibitor in the IMvigor210 cohort. **(E)** The fractions of responses to anti–PD-L1 inhibitor in high and low immune score groups in the IMvigor210 cohort. **(F)** Kaplan–Meier analyses for patients with high or low immune score in the IMvigor210 cohort.

We collected genomic and clinical data of two immunotherapeutic cohorts. Figure 7A visualizes the diverse therapeutic responses to anti–PD-1 inhibitor in the cohort of Liu et al. Especially, we found that low immune score group had the higher fraction of response to anti–PD-1 immunotherapy (Figure 7B). High immune score was linked with more dismal clinical prognosis (Figure 7C). In addition, distinct therapeutic responses and clinical outcomes were investigated between high and low immune score groups in the IMvigor210 cohort (Figures 7D–F).

## Discussion

OC is an aggressive epithelial malignancy, which represents the main cause of cancer morbidity and mortality among females (Matei and Nephew, 2020). Therapeutic options of OC are of limited clinical benefits and adversely influence patients’ quality of life, which represent an unmet need for tolerable effective therapies. Immuno-oncology regimens, which reverse the immune-suppressive tumor microenvironment, may unleash the immune system against cancer cells (Iyer et al., 2021). Hence, determining diverse immune subtypes in the tumor immune microenvironment might offer an insight into the antitumor immune responses as well as promote more effective precision immunotherapeutic regimens.

This study conducted two immune subtypes in accordance with prognostic immunologically relevant genes through consensus clustering analyses. Especially, immune subtype 2 presented more dismal clinical prognosis than immune subtype 1. Cancer is a malignancy triggered by genomic variations and mutations (Kanchi et al., 2014). Immune subtype 1 was characterized by more frequent genetic mutations. A few genomic mutations, such as TP53, are correlated to immunotherapeutic efficacy and possess predictive potential (Sun et al., 2020). TP53 mutation was the first mutated gene across OC. Compared with immune subtype 2, TP53 presented higher mutated frequency in immune subtype 1. Our evidences indicate the difference in genomic variations and mutations between immune subtypes.

Tumor-infiltrating lymphocytes in the tumor microenvironment present correlations to OC outcomes, and immune evasion mechanism is linked with dismal prognosis (Ghisoni et al., 2019). Immune responses are orchestrated by diverse immune cell subpopulations and immune checkpoint molecules (Wang et al., 2021; Zhang et al., 2021). Accumulating evidences suggest that immune infiltrates are correlated to immunotherapeutic responses of OC patients (Yang et al., 2021). Our data demonstrate that immune subtype 1 presented the features of enhanced immune infiltrates and increased expression of MHC molecules. Few predictive markers such as PD-L1 expression and TMB in tumor cells might enable OC positioning as well as patients’ risk stratification (Bi et al., 2020; Wan et al., 2021). Herein, our evidences show that immune subtype 1 was linked with increased TMB score among OC patients. Anti–PD-1/PD-L1 inhibitors have presented the favorable efficacy against diverse cancer types but merely can reach modest objective responses against relapsed OC patients (Westergaard et al., 2020). Immune subtype 1 showed remarkably enhanced expression of PD-1 and PD-L1 than immune subtype 2, indicating that patients in immune subtype 1 presented higher possibilities to respond to anti–PD-1/PD-L1 therapy.

Here, we screened 499 immune subtype–derived genes that might modulate immunity-relevant biological processes and signaling, indicative of their critical roles in tumor immunity. With combination of Boruta feature importance analyses and univariate Cox regression models, 30 prognosis-relevant characteristic immune subtype–derived genes were identified for developing an immune scoring system through PCA algorithm. Further analyses uncovered that high immune score predicted more dismal clinical prognosis and progression of OC patients. In addition, we noticed that high immune score was linked with reduced immune infiltrates and MHC molecule expression. Hence, this immune score may reflect preexisting antitumor immunity of OC. In two anti–PD-1/PD-L1 therapy cohorts, we evaluated the capacities of immune score in estimating therapeutic responses. Our findings indicate that immune score presented favorable efficacy in predicting the benefits from anti–PD-1/PD-L1 therapy.

Initial chemotherapy is effective, but most patients experience chemoresistance (Jordan et al., 2020). Chemoresistance occurs because of the presence of subpopulations of dormant tumor cells within the tumor mass, which facilitate and maintain tumor growth as well as trigger chemoresistance, contributing to relapse following chemotherapeutic agents (Chang et al., 2020). Evidences have demonstrated that immune/inflammatory signals exert prominent functions in chemoresponse or chemoresistance (Jordan et al., 2020). Herein, high immune score indicates enhanced sensitivity to doxorubicin and vinorelbine as well as reduced sensitivity to cisplatin, demonstrating that this immune score possessed the potential in estimating the responses of OC patients to chemotherapeutic agents (doxorubicin, vinorelbine, and cisplatin). Our results uncovered that understanding the tumor immunity allowed us to overcome chemoresistance as well as ameliorate patients’ clinical prognosis (Ghoneum et al., 2021).

The well-defined immune score possessed several advantages than conventional prognostic signatures in OC. First, this immune score might be used to compare diverse immune modulatory factors as well as to investigate the interactions of tumor cells with immune microenvironment. Second, it assists in stratifying OC patients into diverse subpopulations who are suitable for distinct immune checkpoint blockades or benefit from chemotherapy. Moreover, our immune subtype and immune score might facilitate the genomic analyses of genotype–immunophenotype interactions, which is critical for improving the understanding about immunogenomic profiles of OC.

## Conclusion

Collectively, our study characterized the immune subtypes of OC from an immunogenomic perspective. Moreover, we conducted immune score for assessing the immune status and predicting clinical prognosis and therapeutic benefits of OC patients, which might be significant for the stratification of individuals in immunotherapy clinical trials.

## Data Availability

The original contributions presented in the study are included in the article/Supplementary Material, further inquiries can be directed to the corresponding authors.

## Abbreviations

OC, ovarian carcinoma; TCGA, the cancer genome atlas; TPM, transcript per million; CNV, copy number variation; SNP, single-nucleotide polymorphism; MSigDB, molecular signatures database; OS, overall survival; HRs, hazard ratios; ssGSEA, single-sample gene set enrichment analyses; ESTIMATE, estimation of STromal and Immune cells in MAlignant tumor tissues using expression; TMB, tumor mutation burden; PCA, principal component analyses; PCs, principal components; KEGG, Kyoto Encyclopedia of Genes and Genomes; GO, gene ontology; GDSC, Genomics of Drug Sensitivity in Cancer; IC_50_, half-maximal inhibitory concentration; t-SNE, t-distributed stochastic neighbor embedding; OS, overall survival; DSS, disease-specific survival; PFI, progression-free interval.
